# Skin Aging: From Molecular Mechanisms to Therapeutic and Technological Innovations

**DOI:** 10.1111/jcmm.71357

**Published:** 2026-09-20

**Authors:** Sanyin Chen, Yi Yang, Xiaohua Pan, Yu Pan, Sa Cai

**Affiliations:** ^1^ Medical School, Shenzhen University Shenzhen China; ^2^ Laboratory of Regenerative Medicine The 2nd Affiliated Hospital of Shenzhen University Shenzhen China

**Keywords:** anti‐aging, artificial intelligence, delivery systems, senotherapeutics, skin aging, stem cell therapy

## Abstract

Aging is a complex, pervasive biological process that causes irreversible declines in function across organ systems. The skin, the body's largest organ, interfaces directly with the external environment and undergoes aging driven by intrinsic (chronological) and extrinsic (environmental) factors, which result in functional deterioration and greater susceptibility to related disorders. Although numerous anti‐aging strategies have been investigated, relatively few specifically target the skin. This review delineates the principal phenotypes and underlying mechanisms of skin aging. Phenotypic hallmarks include reduced regenerative capacity, chronic inflammation and impaired barrier function, whereas key mechanisms comprise oxidative stress, DNA damage, paracrine induction of cellular senescence and epigenetic alterations. The review evaluates recent research advances and associated clinical challenges in mitigating skin aging, with particular emphasis on pharmacological interventions and stem cell therapy. Finally, it briefly reviews delivery systems for natural anti‐aging compounds and explores the potential of artificial intelligence (AI) to accelerate novel drug discovery.

## 1 Introduction

The global population is aging significantly; by 2050, individuals aged 60 years and older are projected to comprise more than 20% of the population [1]. Aging is a gradual, irreversible pathophysiological process that impairs physiological functions across organ systems and increases mortality risk. It contributes to declines in respiratory and renal function, a higher incidence of cardiovascular disease and reductions in skeletal muscle mass and strength [2, 3, 4, 5]. The physiological signs of aging are particularly pronounced in the skin.

Skin aging is a multifactorial process driven by intrinsic and extrinsic factors. Intrinsic aging refers to physiological changes that occur over time, typically manifested as skin atrophy, thinning, dryness and fine lines [6]. The aging of unexposed skin areas is primarily linked to genetic and metabolic factors [6]. In contrast, extrinsic aging results from environmental exposures such as ultraviolet (UV) radiation, air pollution and smoking [6]. Among these, UV radiation is the predominant factor, responsible for approximately 80% of visible signs of skin aging [7, 8]. UV radiation chiefly comprises UVB (280–320 nm) and UVA (320–400 nm); UVB predominantly affects the epidermis and can directly damage DNA, whereas UVA penetrates more deeply to the dermis and induces oxidative damage through the generation of reactive oxygen species (ROS) [9]. UV‐induced aging is commonly termed photoaging [7, 8]. A key characteristic of photoaging is the abundance of pathologically altered elastic fibres, often termed ‘solar elastosis’ [10].

Importantly, skin aging contributes to a range of local and systemic disorders. With advancing age, the skin becomes increasingly vulnerable to pruritus, fungal infections and cutaneous malignancies [11]. Aged skin also exhibits elevated levels of pro‐inflammatory cytokines, which have been linked to systemic conditions such as psoriasis, atopic dermatitis, obesity and diabetes [12]. Moreover, the accumulation of senescent cells in the skin has been linked to brain disorders and cognitive decline [13]. Consequently, it is essential to implement effective strategies to mitigate the progression of skin aging.

The development of drugs focused on rejuvenation has emerged as a popular research area. Currently, several anti‐aging agents have exhibited substantial potential for delaying aging, prolonging lifespan and alleviating various disease symptoms [14, 15, 16, 17, 18, 19]. These agents may be considered viable candidates for addressing skin aging. Additionally, due to the limited availability of drugs, a novel approach is necessary to enhance the efficiency of new drug screening.

Here, we review the characteristics of skin aging, the roles of various types of senescent cells and related mechanisms underlying skin aging. Then, recent advancements on approaches to treat skin aging and their associated challenges are discussed respectively. In addition, the application of AI in the field of aging is briefly explored.

## 2 The Influence of Various Types of Senescent Cells on Skin Aging

**FIGURE 1:**
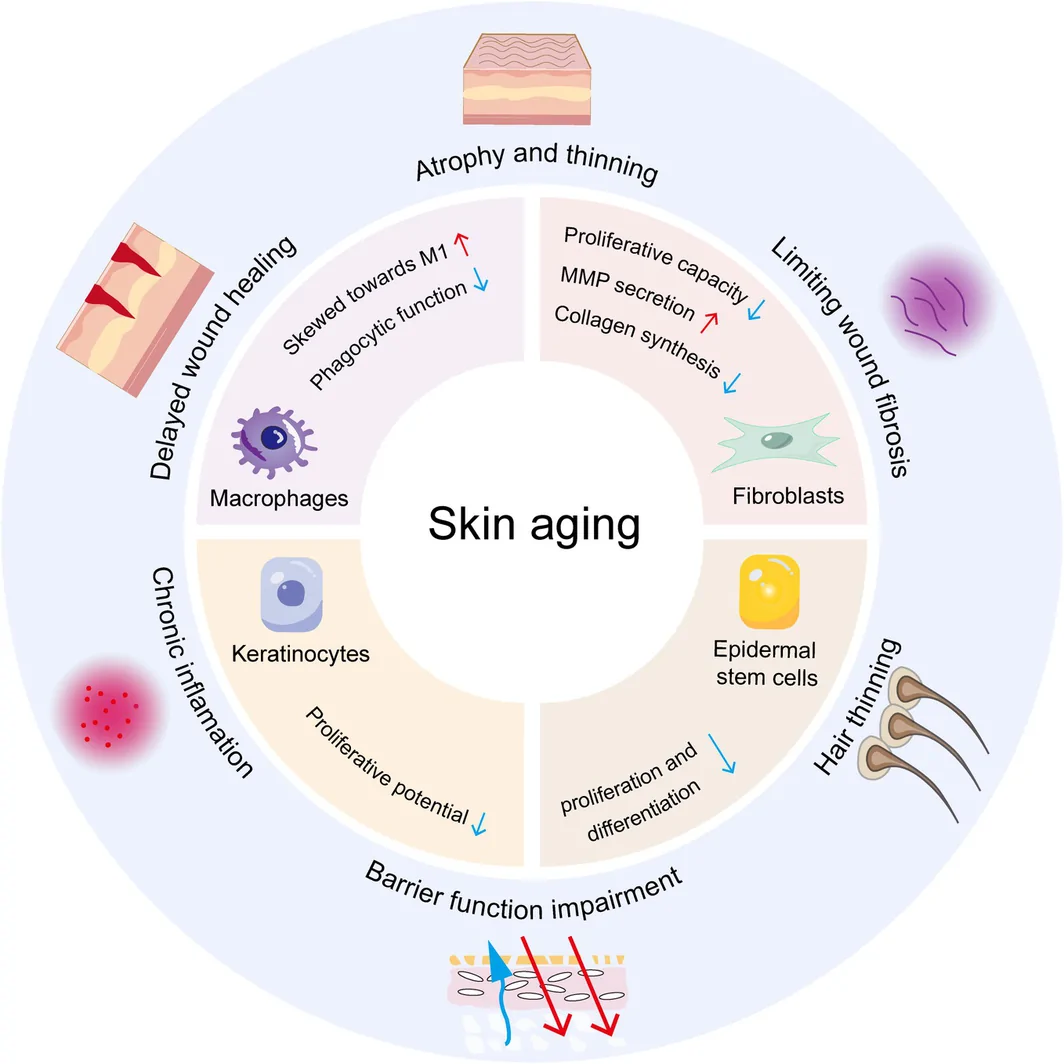
Major phenotypic features of skin aging and functional alterations in principal skin cell types.

Cells can undergo senescence as a result of various stressors. Because senescent cells resist apoptosis and are inefficiently cleared by the immune system, they progressively accumulate in tissues [17, 20]. Functional changes in senescent skin cells contribute to aging phenotypes, including reduced regenerative capacity, chronic inflammation, impaired barrier function, and skin thinning and wrinkling (Figure 1). In skin, the principal senescent cell types are fibroblasts, keratinocytes, macrophages and epidermal stem cells.

### 2.1 Effects of Senescent Fibroblasts on Wound Healing, Fibrosis and Dermal Integrity

Fibroblasts are the predominant cell type in the dermis. They display robust proliferative and migratory capacities, enabling rapid responses to injury and active participation in wound healing and tissue regeneration [21]. Fibroblasts also synthesize essential structural proteins, including Type I collagen, Type III collagen and elastin, which are vital for maintaining dermal architectural integrity [22]. However, excessive accumulation of senescent fibroblasts impairs wound healing, promotes dermal atrophy, reduces elasticity and restricts fibrosis.

Fibroblast senescence exerts a dual effect on cutaneous wound healing. In young mice, transient upregulation of fibroblast senescence promotes the differentiation of myofibroblasts through the secretion of Platelet‐Derived Growth Factor AA (PDGF‐AA), thereby facilitating optimal wound closure [23, 24]. As aging progresses, the increase in p21‐positive fibroblasts in mouse skin leads to a reduction in cell proliferation capacity and delays wound repair [25]. Furthermore, senescent cells establish a senescence microenvironment through paracrine signalling, further delaying the wound healing process [26, 27]. Consequently, targeted clearance of senescent cells may enhance the repair of aged wounds.

Fibroblasts maintain the skin's structural integrity by dynamically regulating the extracellular matrix (ECM). Cellular senescence disrupts the balance between ECM synthesis and degradation. In the dermis, senescent fibroblasts decrease production of ECM components, which is associated with impairment of key ECM‐synthesis signalling pathways (Figure 1). They also promote dermal degradation by secreting matrix metalloproteinases (MMPs), the principal mediators of ECM breakdown [28] (Figure 1). Together, these changes reduce ECM content and contribute to skin wrinkling and decreased elasticity.

Myofibroblasts are specialized fibroblasts with smooth muscle–like characteristics that differentiate from activated fibroblasts [29]. Fibrosis, defined by excessive ECM deposition, is primarily driven by fibroblasts and myofibroblasts [30, 31]. Senescent myofibroblasts contribute to limiting wound fibrosis because they exhibit reduced proliferative capacity, diminished ECM synthesis and increased ECM degradation [32]. Moreover, senescent myofibroblasts impair wound contraction and delay healing by reducing expression of α‐smooth muscle actin (α‐SMA), a key contractile protein [33].

### 2.2 Effects of Senescent Macrophages on Skin Inflammation, Wound Healing and Structural Integrity

Macrophages exhibit potent phagocytic activity that clears cellular debris, pathogens and other unnecessary wastes. In addition, they play a crucial role in regulating inflammation, wound healing and dermal structural remodelling [34]. Macrophage senescence results in chronic skin inflammation, impaired wound healing, wrinkle formation and reduced tissue elasticity.

Macrophages are generally classified into pro‐inflammatory M1 and anti‐inflammatory M2 subtypes [35], although an expanding spectrum of activation states and subtypes has been recognized [36]. M1 macrophages release pro‐inflammatory factors, including TNF‐α, IL‐1β and IL‐12, to neutralize stimuli such as microorganisms that may infiltrate the skin wound [37]. In the early stage of wound healing, M1 cells protect the wound by clearing foreign substances and phagocytosing necrotic tissue and pathogens [38, 39]. M2 macrophages suppress the inflammatory cascade by releasing IL‐10 and TGF‐β, thereby promoting transition to the tissue repair phase [37]. A decline in proliferative and phagocytic capacity in aged macrophages leads to prolonged inflammation and delayed healing [40, 41]. Moreover, increased polarization toward the M1 phenotype and elevated pro‐inflammatory gene expression in aged macrophages exacerbate tissue damage and impair healing in aged wounds [37, 42, 43].

Additionally, a study has demonstrated that the skewing of M1/M2 macrophages influences collagen abundance in aged skin [44]. The reduction of M2 macrophages is associated with a decrease in the synthesis of collagen types I, V and VI, while the upregulation of M1 macrophages exacerbates the degradation of dermal collagen by increasing the expression of MMPs [44]. Thus, senescent macrophages may act synergistically with senescent fibroblasts to drive dermal thinning, wrinkling, and increased fragility in aged skin.

### 2.3 Effects of Keratinocyte Senescence on Skin Barrier Function and Wound Healing

Keratinocytes are the predominant cell type in the human epidermis and are responsible for forming and maintaining the epidermal barrier [45]. They have robust proliferative and migratory capacities, enabling rapid re‐epithelialization following injury [46]. Keratinocyte senescence impairs barrier function and delays wound healing.

Diminished proliferative capacity of senescent keratinocytes slows epidermal renewal in older individuals, resulting in epidermal thinning [47, 48]. This thinning increases moisture loss in elderly skin [49]. Chronic reduction of stratum corneum hydration and declined epidermal barrier function render aged skin more susceptible to inflammatory dermatoses and other chronic systemic conditions [50]. Barrier impairment also facilitates cutaneous colonization by pathogens such as *Staphylococcus aureus* [51]. Additionally, the diminished proliferative and migratory capacity of senescent keratinocytes delays wound healing and contributes to age‐related chronic wounds [52].

### 2.4 Effects of Senescent Epidermal Stem Cells on Wound Healing and Hair Growth

Epidermal stem cells (ESCs) are primarily located in the basal layer and the hair follicle bulge and can be categorized into infundibular epidermal stem cells (IFESCs) and hair follicle stem cells (HFSCs) [53]. ESCs exhibit self‐renewal and differentiation capacities that enable continuous replenishment of epidermal cells and maintenance of skin homeostasis [53].

Robust activation of ESCs and efficient recruitment of their progeny to the epidermal lineage are crucial for re‐epithelialization of skin wounds [54]. However, ESC senescence markedly impairs wound healing [55]. During epidermal aging, reduced expression of peroxisome proliferator‐activated receptor gamma coactivator 1‐alpha (PGC‐1α) delays wound healing in mice by impairing ESC proliferation and differentiation [55].

Hair growth involves the transition of HFSCs from a quiescent phase to an active growth phase [56]. During the hair cycle, the behaviours of HFSCs are regulated by various transcription factors [56]. Among these, Nfatc1 is essential for maintaining HFSC quiescence. An age‐related decline in SIRT7 impairs Nfatc1 deacetylation, delays HFSC activation and contributes to hair thinning—a prominent marker of skin aging [57].

## 3 Mechanisms of Skin Aging

The mechanisms underlying skin aging exhibit several similarities to those of systemic aging, including epigenetic alterations and chronic inflammation [58]. Meanwhile, the skin is especially vulnerable to UV radiation and air pollution. Consequently, the resultant DNA damage and oxidative stress are primary factors that induce cellular senescence in the skin [59, 60]. Furthermore, the accumulation of senescent skin cells establishes a senescent microenvironment through the senescence‐associated secretory phenotype (SASP), which further induces skin cell senescence and the age‐associated alterations in skin tissues.

### 3.1 ROS and DNA Damage

Under normal physiological conditions, ROS are natural byproducts of cellular metabolism, predominantly generated during mitochondrial oxidative phosphorylation. Both intrinsic mitochondrial dysfunction and external UV exposure contribute to the increased production of ROS [61]. Excessive ROS levels are a principal driver of skin aging.

**FIGURE 2:**
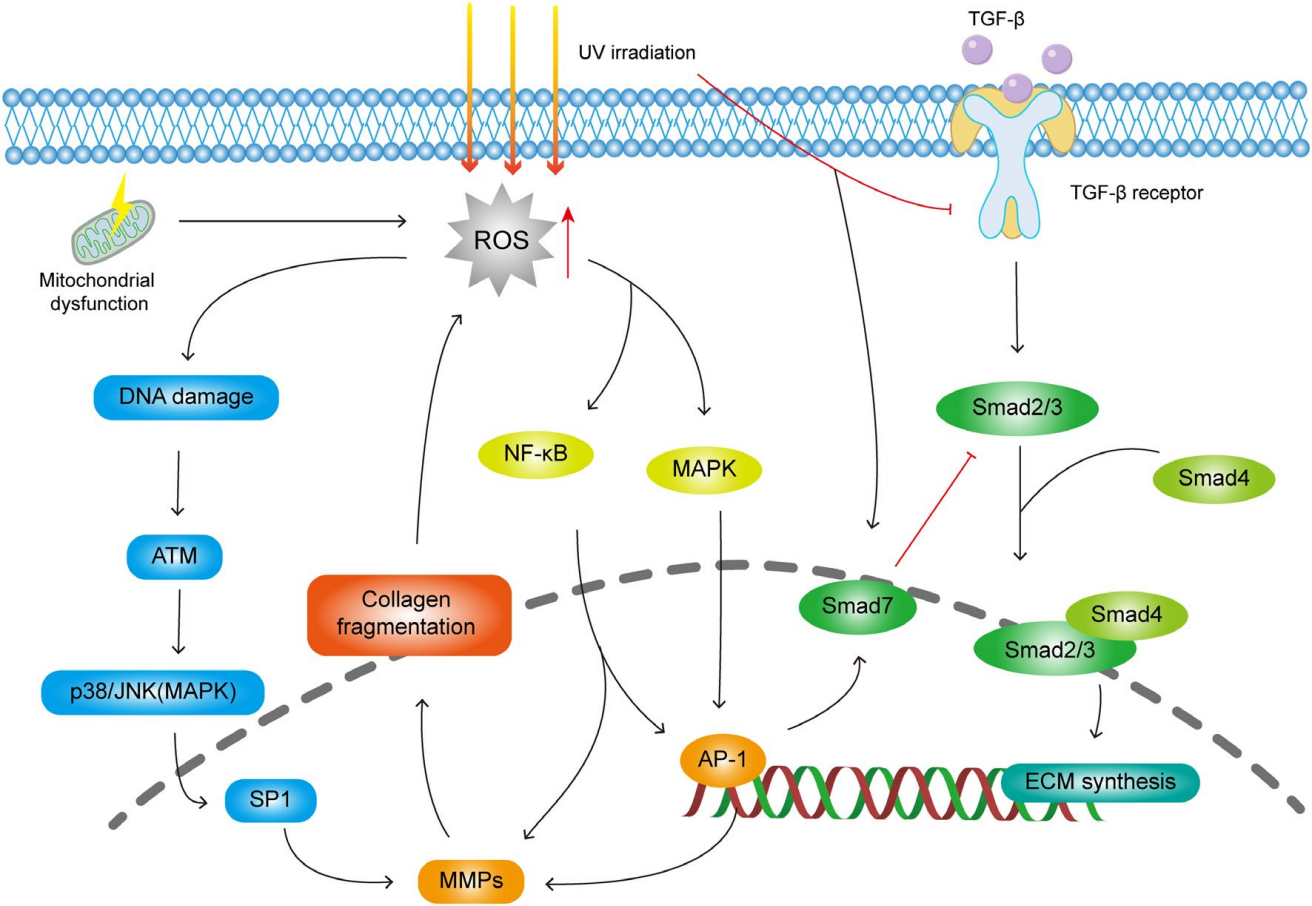
Major mechanisms regulating ECM synthesis and degradation in aged skin. Mitochondrial dysfunction and UV irradiation increase intracellular ROS. Excess ROS activate the NF‐κB and AP‐1, promoting overexpression of MMPs. ROS also induce DNA damage and, via the ATM/MAPK pathway, activate Sp1, further enhancing MMP expression. MMPs mediate the degradation of dermal collagen and compromise ECM integrity. Consequently, the mechanical resistance of the ECM declines, weakening the mechanical tension experienced by fibroblasts and ultimately leading to further increases in intracellular ROS levels. The TGF β/Smad signalling pathway is the primary driver of dermal ECM synthesis. UV radiation and AP‐1 inhibit this pathway by downregulating TGF‐β receptors and upregulating the inhibitory regulator Smad7, ultimately reducing ECM production.

In senescent cells, ROS induce the activation of activator protein 1 (AP‐1) through the mitogen‐activated protein kinase (MAPK) and nuclear factor kappa‐light‐chain‐enhancer of activated B cells (NF‐κB) signalling pathways [62]. AP‐1 is a heterodimeric transcription factor composed of c‐Fos and c‐Jun; its activation leads to the degradation of collagen and elastin by increasing the expression of MMPs [62]. Concurrently, AP‐1 reduces synthesis of ECM components, further contributing to dermal atrophy [63] (Figure 2). Additionally, NF‐κB directly promotes MMP transcription and expression [62]. Notably, the accumulation of MMP‐induced fragmented collagen in the dermis reduces the mechanical resistance of the ECM and consequently decreases intracellular mechanical tension in fibroblasts; this alteration in mechanotransduction impairs fibroblast function, further increases ROS production, and establishes a positive feedback loop that accelerates skin aging [64, 65].

In addition, ROS directly damage DNA, causing base oxidation, deoxyribose modification and strand breaks [60, 66]. When DNA damages are recognized, DNA repair pathways are activated to restore genomic integrity, including nucleotide excision repair (NER), base excision repair (BER) and non‐homologous end joining (NHEJ) [67, 68]. However, excessive ROS can damage the cellular DNA repair proteome and compromise DNA repair [69].

Persistent DNA damage signalling can drive cells into an irreversible senescent state, accompanied by the formation of the SASP [70]. DNA damage activates the ataxia‐telangiectasia mutated (ATM) and ATM‐ and Rad3‐related (ATR) kinases, which in turn activate checkpoint kinases CHK1 and CHK2 and thereby induce the p53–p21 signalling pathway, culminating in permanent cell cycle arrest and cellular senescence [67]. DNA damage also activates NF‐κB signalling via an ATM‐NEMO‐dependent regulation of an upstream kinase, thereby promoting secretion of SASP factors [67]. Additionally, ATM activates the transcription factor SP1 through the MAPK pathway, and SP1 strongly induces expression of MMP‐2 and MMP‐11 [71] (Figure 2).

### 3.2 The Senescent Environment in Skin

The SASP secreted by senescent cells constitutes a crucial element of the senescent microenvironment [70]. It serves as a critical mediator of intercellular communication, transmitting senescence signals to neighbouring cells [72]. SASP comprises a variety of bioactive molecules, including inflammatory cytokines, growth factors and extracellular vesicles (EVs) [73, 74, 75].

#### 3.2.1 TGF‐β

TGF‐β is a multifunctional cytokine that belongs to the TGF‐β superfamily [76]. It plays a crucial role in regulating cell proliferation, cellular senescence and ECM synthesis [76, 77, 78].

In skin aging, dermal fibroblasts produce and release TGF‐β [79]. TGF‐β binding to its receptors activates Smad2/3, which forms a heterotrimeric complex with Smad4 [31, 80]. The Smad2/3–Smad4 complex is the core transcriptional effector of the TGF‐β pathway and regulates transcription of genes associated with the cell cycle and ECM components [78, 80] (Figure 2). TGF‐β induces cell cycle arrest and senescence by upregulating cyclin‐dependent kinase (CDK) inhibitors (p15, p21, p27) [80, 81, 82]. During photoaging, reduced TGF‐β receptor expression in the dermis decreases TGF‐β/Smad‐mediated collagen synthesis [83]. Additionally, UV radiation induces the expression of inhibitory Smad7, which limits ECM deposition by antagonizing Smad2/3 signalling [84, 85].

#### 3.2.2 IL‐1α

IL‐1α, a significant pro‐inflammatory cytokine within the IL‐1 family, is constitutively expressed by keratinocytes and is retained as an intracellular store [86]. UV irradiation induces keratinocytes to release IL‐1α, promoting cellular senescence and triggering skin inflammation [87, 88].

**FIGURE 3:**
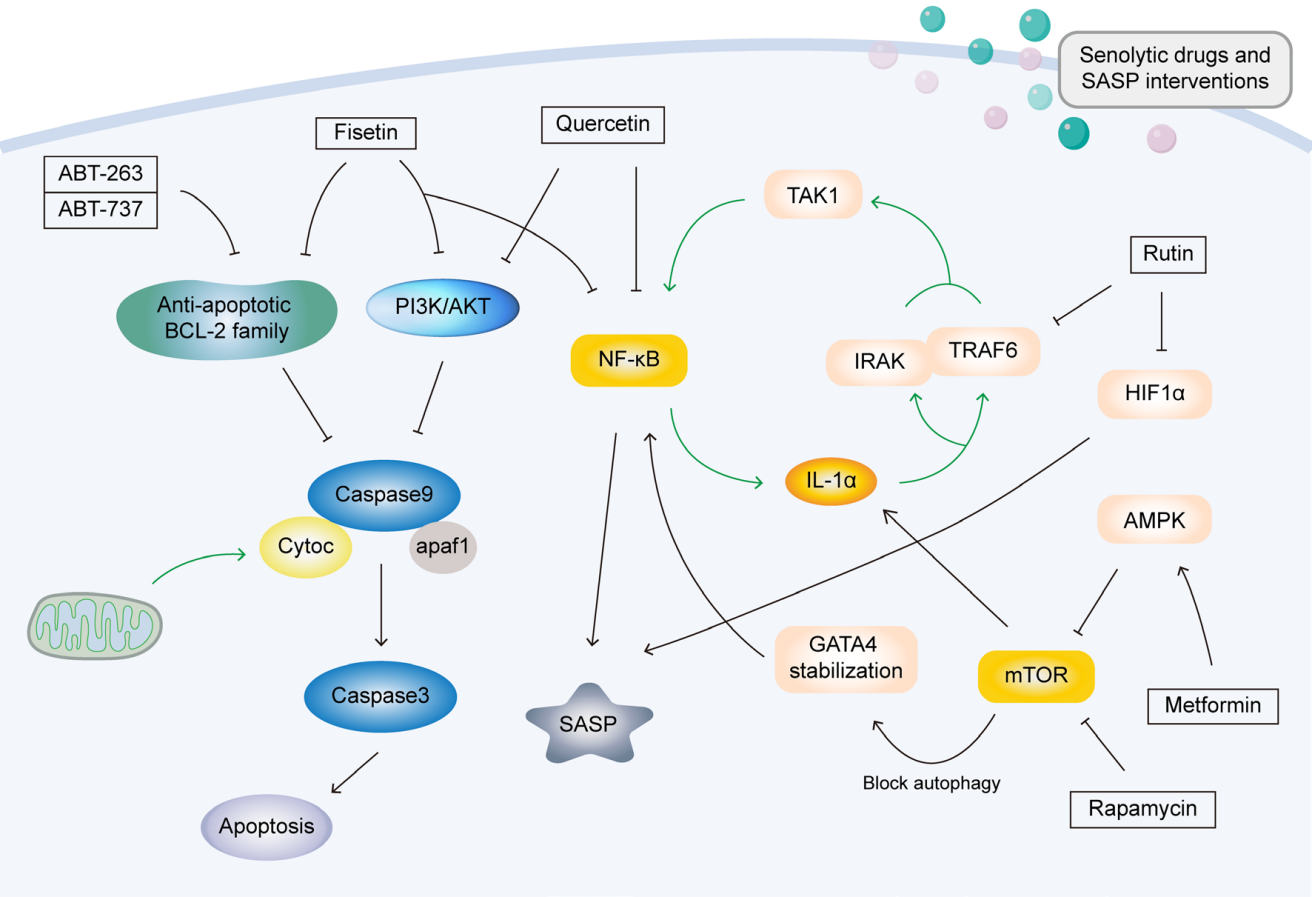
Senotherapeutics delay skin aging by eliminating senescent cells and modulating the SASP. Senolytic agents (ABT‐263, ABT‐737, fisetin, quercetin) promote skin rejuvenation by inducing apoptosis of senescent cells, primarily by inhibiting anti‐apoptotic BCL‐2 family proteins and suppressing the PI3K/AKT signalling pathway, thereby relieving inhibition of the caspase‐9‐dependent apoptotic cascade. Modulating the SASP also mitigates skin aging. Major SASP regulators include NF‐κB, IL‐1α and mTOR; direct or indirect inhibition of these factors can attenuate the negative effects of the SASP on skin.

Binding of IL‐1α to the receptor IL‐1R1 initiates a signalling cascade that modulates inflammation. After IL‐1α engages IL‐1R1, it recruits the IL‐1 receptor accessory protein (IL‐1RAcP) to form a heterodimeric receptor complex that activates IL‐1 receptor‐associated kinase (IRAK). IRAK is the principal downstream mediator of IL‐1α–induced inflammation [89]. IRAK collaborates with Tumour Necrosis Factor Receptor‐Associated Factor 6 (TRAF6) to activate TAK1, which in turn triggers the NF‐κB pathway and thereby promotes the expression of downstream SASP factors, notably IL‐6 and IL‐8 [89, 90]. Concurrently, TAK1 activates MAPK, further enhancing the inflammatory response [89]. Importantly, NF‐κB activation also upregulates IL‐1α expression [91], forming a positive feedback loop that can amplify inflammation and senescence (Figure 3). Consequently, IL‐1α represents a potential therapeutic target to mitigate skin inflammation and aging.

#### 3.2.3 EVs

EVs are membrane‐enclosed vesicles released by cells, containing various components, such as DNA, non‐coding RNAs, membrane lipids and proteins [92]. After uptake by diverse recipient cells, EV cargo can modulate recipient cell pathophysiology, including cellular senescence [93, 94, 95]. Notably, senescent cells release more EVs than young cells, suggesting a greater impact on neighbouring cells [96].

UV radiation induces DNA strand breaks in skin cells and leads to the formation of nuclear DNA fragments [97, 98]. Skin cells may expel toxic DNA fragments by increasing the release of senescence‐associated EVs [99, 100, 101]. Consequently, neighbouring non‐senescent cells may be affected by EVs carrying DNA fragments. Siametis et al. showed that EVs from TFIIS‐deficient mouse embryonic fibroblasts (MEFs) containing TelC‐DNA fragments induce senescence in recipient MEFs, potentially via activation of the cGAS‐STING signalling pathway [102]. Activation of the cGAS–STING pathway induces the expression of SASP genes by activating inflammation‐associated transcription factors, including IRF3, NF‐κB and STAT6 [99, 103]. These findings suggest that EV‐mediated transfer of toxic nuclear DNA fragments may represent a novel mechanism contributing to skin aging.

MicroRNAs (miRNAs) typically interact with the 3′ untranslated regions (3′ UTR) of target mRNAs, leading to mRNA degradation or translation repression and thereby impacting cellular biological events [104]. EV‐mediated transfer of miRNAs can induce senescence in skin cells. EV‐associated miR‐30a is a key regulator of human epidermal aging because it suppresses genes including LOX, which regulates the proliferation–differentiation balance of keratinocytes [105, 106]. miR‐30a carried by EVs from senescent keratinocytes impairs the proliferation, differentiation and migration of recipient young keratinocytes [105]. miR‐326‐3p is enriched in EVs derived from senescent endothelial cells and regulates proliferation and senescence of skin fibroblasts [107]. Mechanistically, miR‐326‐3p promotes fibroblast senescence by targeting and downregulating Toll‐like receptor 4 (TLR4) expression [107], and TLR4 has been implicated in inflammation and aging [108]. Additionally, EV‐associated miR‐10a, miR‐30c and miR‐451a increase ROS by modulating mitochondrial function, contributing to dermal aging [109]. Collectively, these miRNAs represent potential therapeutic targets for skin aging.

The roles of other components encapsulated in EVs in skin aging require further investigation. Moreover, owing to their cargo capacity, EVs may serve as delivery vehicles to transport bioactive agents for anti‐aging interventions in the skin.

### 3.3 Regulation of Macrophage Polarization by Histone Modifications

**FIGURE 4:**
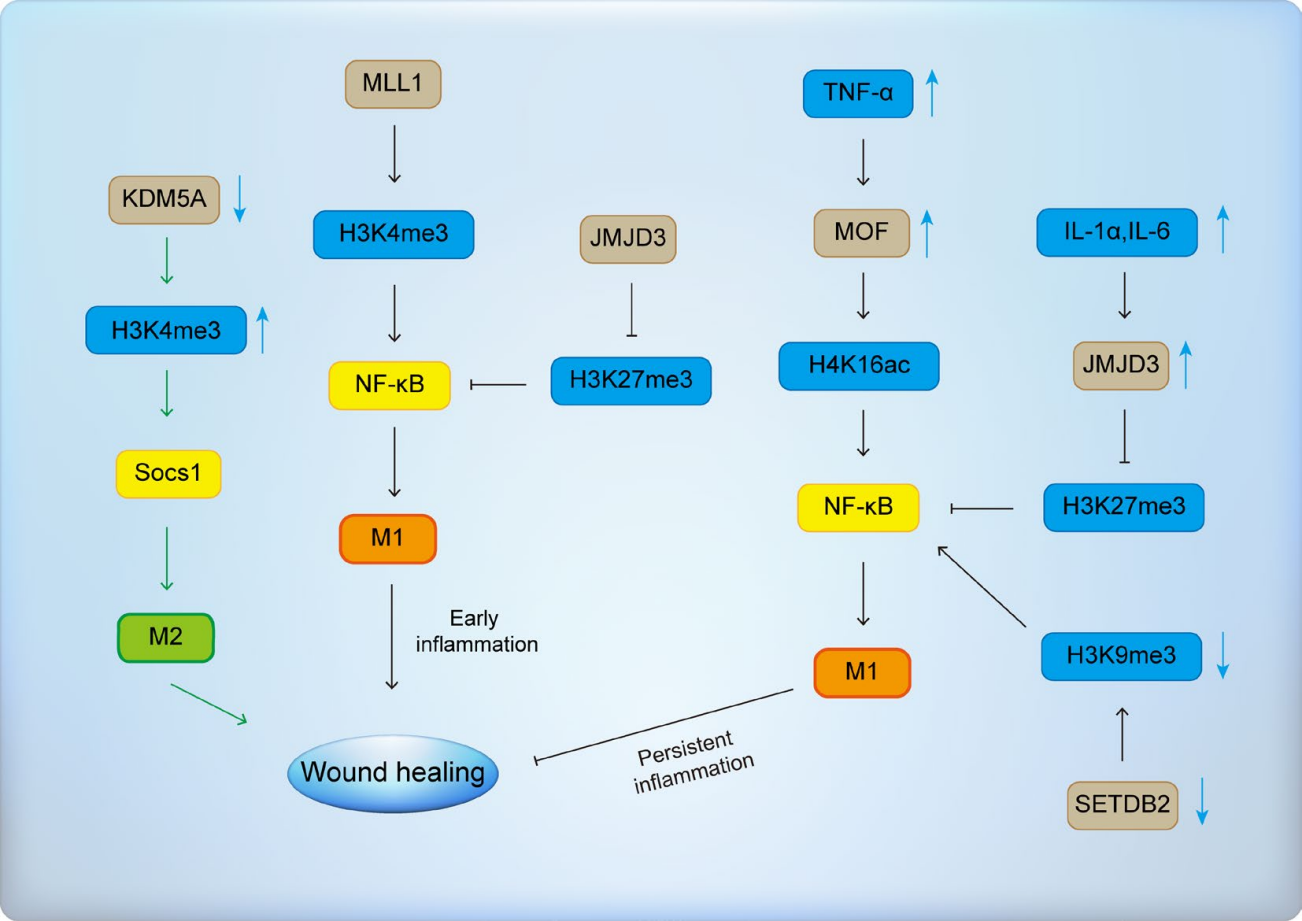
Major histone modifications regulate macrophage M1/M2 polarization during skin wound healing. Several epigenetic regulators remodel histone marks to regulate macrophage polarization toward pro‐inflammatory M1 or reparative M2 phenotypes. During the early inflammatory phase, JMJD3 and MLL1 are upregulated, promoting M1 polarization and facilitating tissue repair. Conversely, timely downregulation of KDM5A favours M2 polarization. Under stimulation by pro‐inflammatory cytokines (IL‐1α, IL‐6, TNF‐α), JMJD3 and MOF remain highly expressed, sustaining transcription of inflammatory genes and thereby prolonging the inflammatory phase and delaying healing. Thus, in aged skin, sustained upregulation of these pro‐inflammatory regulators—manifested as persistent JMJD3 and MOF expression—may impede wound repair by preventing resolution of inflammation and transition to the M2 phenotype. Furthermore, downregulation of SETDB2 promotes M1 polarization and increases inflammatory gene expression.

The polarization of M1 and M2 macrophages is essential for regulating the inflammatory response during skin wound healing. Epigenetic modifications, particularly histone modifications, play a significant role in modulating macrophage polarization [110] (Figure 4). Aging may affect macrophage polarization in part by changing histone modification patterns, including methylation, demethylation, acetylation and lactylation.

H3K4me3 is an active mark catalysed by the mixed lineage leukaemia‐1 (MLL1) methyltransferase and is closely associated with macrophage polarization during wound healing [111]. During the early wound stage, elevated MLL1 increases H3K4me3 and thereby enhances NF‐κB activity, promoting production of pro‐inflammatory cytokines. Conversely, during the late inflammatory phase, downregulation of MLL1 suppresses NF‐κB activity, facilitating macrophage transition to the anti‐inflammatory M2 phenotype [111]. Additionally, downregulation of the histone demethylase KDM5A is required for M2 polarization during murine skin wound healing [112]. KDM5A deficiency increases H3K4me3 at the promoter of suppressor of cytokine signalling 1 (Socs1), a negative regulator of immune responses, thereby promoting M2 polarization and accelerating wound healing [112]. Currently, no studies have reported that persistent expression of MLL1 and KDM5A in aged wounds contributes to delayed healing.

H3K27me3 is a repressive histone modification, and the Jumonji domain‐containing protein 3 (JMJD3; also known as KDM6B) activates gene expression by demethylating H3K27me3 [113]. Demethylation of H3K27me3 by JMJD3 plays a critical role in modulating macrophage inflammatory responses. Audu et al. showed that JMJD3 upregulation in macrophages increases IL‐1β and TNF‐α expression by reducing H3K27me3, thereby driving the early inflammatory response in wounds. However, during the late inflammatory phase in diabetic wounds, sustained JMJD3 upregulation drives persistent NF‐κB–mediated transcription of inflammatory genes, ultimately impairing wound healing [114]. Moreover, elevated IL‐1α and IL‐6 in diabetic wounds induce JMJD3 overexpression [114, 115]. Thus, IL‐1α and IL‐6 may impede macrophage polarization from M1 to M2 phenotype in aged wounds by promoting JMJD3 overexpression (Figure 4).

H3K9me3 is a repressive mark specifically catalysed by the methyltransferase SET domain bifurcated 2 (SETDB2) [116, 117]. In human and murine wound macrophages, SETDB2‐mediated H3K9me3 represses NF‐κB‐dependent inflammatory gene expression and promotes polarization toward an M2 phenotype [118]. By contrast, in diabetic wound tissue, downregulation of SETDB2 increases macrophage inflammatory gene expression and delays wound healing [118]. To clarify the regulatory role of H3K9me3 in immune senescence, analysis of its genomic localization and abundance in senescent macrophages is essential.

Males absent on the first (MOF; also known as KAT8/MYST1), a histone acetyltransferase, acetylates histone H4 at lysine 16 (H4K16ac) and thereby promotes gene transcription [119]. Aaron et al. reported that persistently elevated MOF in diabetic wounds sustains NF‐κB‐mediated inflammatory gene expression via H4K16ac, thereby hindering the transition of macrophages from an inflammatory to a reparative phenotype [120]. Moreover, the study indicated that elevated TNF‐α upregulates MOF expression in wound macrophages [120]. Consequently, aged skin exhibits elevated levels of TNF‐α [121], which may impede M2 polarization in aged wounds by promoting MOF overexpression (Figure 4).

H3K9ac, an active histone modification primarily catalysed by the histone acetyltransferase PCAF, is closely associated with macrophage inflammatory activation. Li et al. showed that PCAF promotes M1 polarization and upregulates pro‐inflammatory cytokine expression by inducing H3K9ac [122]. In addition, PCAF acetylates the NF‐κB subunit p65, activating NF‐κB signalling and thereby increasing pro‐inflammatory cytokine secretion [122].

Histone H3 lysine 18 lactylation (H3K18La), a histone mark associated with transcriptional activation, is primarily catalysed by the acetyltransferases p300/CBP and PCAF [123]. The role of H3K18La in inflammation is context‐dependent. Zhang et al. reported enrichment of H3K18La at the promoters of M2‐associated genes, including Arg1 and Vegfa [124]. Conversely, an age‐associated increase in H3K18La activates NF‐κB signalling by enhancing H3K18La occupancy at the promoters of Rela (p65) and Nfkb1 (p50), thereby promoting IL‐6 and IL‐8 expression in microglia [123]. Further studies are needed to elucidate the specific role of H3K18La in regulating inflammation in aged macrophages.

## 4 Therapeutic Strategies for Combating Skin Aging

Senotherapeutics and stem cell therapy are two significant approaches to combating aging. In recent years, both strategies have demonstrated remarkable translational progress in skin rejuvenation.

### 4.1 Senotherapeutics

Senotherapeutics is a promising strategy with substantial potential to delay aging and to treat age‐related diseases [125]. This field comprises two main classes: senolytics and senomorphics. Senolytics selectively eliminate senescent cells by inducing apoptosis, whereas senomorphics mitigate the negative effects of senescent cells on neighbouring tissues by limiting the SASP.

#### 4.1.1 Anti‐Apoptotic BCL‐2 Family Protein Inhibitors

The BCL‐2 protein family comprises pro‐apoptotic members (e.g., BAX and BAK) and anti‐apoptotic members such as BCL‐2, BCL‐XL, BCL‐W and MCL‐1 [126]. Activation of BAX and BAK leads to alterations in mitochondrial outer membrane permeabilization (MOMP), which facilitates the release of cytochrome c into the cytosol and initiates the assembly of apoptosomes. The apoptosome, primarily composed of cytochrome c and Apaf‐1, activates the caspase cascade via caspase‐9, ultimately leading to cellular apoptosis [127, 128] (Figure 3). By contrast, anti‐apoptotic BCL‐2 family proteins inhibit MOMP and thereby prevent apoptosis [127]. Consequently, inhibitors targeting anti‐apoptotic BCL‐2 family proteins may induce apoptosis in senescent cells (Figure 3).

Navitoclax (ABT‐263) is a small molecule inhibitor with a high affinity for BCL‐XL, BCL‐2 and BCL‐W [129], and it is considered a potential therapeutic agent for skin aging. Studies have shown that ABT‐263 selectively eliminates senescent dermal fibroblasts and reduces MMP secretion, thereby improving features of skin aging and promoting wound healing [130, 131, 132]. ABT‐263 also significantly decreases the population of senescent melanocytes and ameliorates aging‐related pigmentary changes [133]. However, Kohli et al. reported that senescent melanocytes in culture and in nevi express elevated levels of BCL‐W but remain insensitive to ABT‐263; this insensitivity is attributed to the upregulation of the anti‐apoptotic protein MCL‐1 in these cells [134]. The combination of ABT‐263 with MCL‐1 inhibitors markedly enhanced therapeutic efficacy in that context [134]. Additionally, ABT‐263 may cause significant side effects, including thrombocytopenia and neutropenia [135]. Further research is needed to assess the safety of ABT‐263 in the context of skin aging treatment.

ABT‐737, like ABT‐263, can eliminate senescent dermal fibroblasts and reduce the levels of SASP in the skin [132]. Other inhibitors, including A‐1331852, A‐1155463 and UBX1967, can facilitate the apoptosis of human umbilical vein endothelial cells (HUVECs), IMR90 cells, and endothelial cells by inhibiting BCL‐XL [19, 136]. These compounds are potential therapeutics for skin aging.

#### 4.1.2 Flavonoids

Natural products are an important source for senotherapeutic development, with flavonoids among the most extensively studied classes [137]. Characterized by their phenolic structures, flavonoids are widely recognized for their beneficial effects on health [138].

Quercetin, a flavonoid primarily found in fruits [139], is a promising compound for delaying aging [140, 141, 142]. It induces apoptosis in senescent cells by inhibiting the phosphoinositide 3‐kinase (PI3K)/protein kinase B (AKT) pathway, thereby reducing phosphorylation of Bad and caspase‐9 [143, 144] (Figure 3). Quercetin is frequently combined with the PI3K/AKT inhibitor dasatinib to enhance senolytic efficacy [143]. Takay et al. reported that the combination of Quercetin and dasatinib significantly improves skin aging by selectively removing senescent dermal fibroblasts [145]. In addition to its senolytic activity, Quercetin inhibits the SASP, exhibits antioxidant properties and regulates M2 polarization. By inhibiting NF‐κB and AP‐1 activity, Quercetin reduces COX‐2 and MMP‐1 expression, mitigating UV‐induced photoaging in human skin [146]. It also promotes M2 polarization and upregulates endogenous antioxidant defences via AMP‐activated protein kinase (AMPK) and AKT signalling, thereby reducing neuroinflammation [147]. Collectively, these findings support quercetin as a candidate for skin‐aging interventions. However, prolonged supplementation has been associated with adverse effects, including mild headaches, tingling sensations, and even severe cardiovascular issues [148], warranting cautious evaluation of safety.

Fisetin is a flavonoid structurally similar to quercetin that exhibits notable anti‐aging activity [18, 149]. As a PI3K/AKT inhibitor, Fisetin promotes apoptosis in senescent cells by inhibiting the PI3K/AKT signalling pathway [143] (Figure 3). Fisetin also downregulates the expression of anti‐apoptotic proteins BCL‐2 and BCL‐W while upregulating the pro‐apoptotic protein BAX, thereby enhancing its apoptotic effects [150] (Figure 3). Additionally, Fisetin significantly reduces the expression of pro‐inflammatory cytokines (TNFα, IL‐1β, IL‐6) and MMPs (MMP‐1, MMP‐3) in skin cells by inhibiting the NF‐κB and MAPK/AP‐1 signalling pathways [151]. Using a mouse/human chimeric model, Takaya et al. reported that fisetin treatment decreases the number of senescent dermal fibroblasts and reduces SASP expression, thereby mitigating skin aging phenotypes [152].

Rutin, also known as vitamin P and quercetin‐3‐O‐rutinoside, is commonly found in various vegetables and is considered a promising senomorphic agent [153, 154]. Rutin reduces expression of IL‐6, IL‐8 and MMPs in senescent cells by inhibiting the activity of the transcription factor HIF1α and the adaptor protein TRAF6 [154] (Figure 3). It also acts as a ROS scavenger, protecting fibroblasts from UVA‐induced damage. Meanwhile, Rutin undergoes oxidation to produce quinone derivatives, which interact with the Keap‐1 protein, thereby activating the Nrf2‐mediated antioxidant system and enhancing endogenous cellular defences [155]. In a randomized, double‐blind clinical trial, topical rutin cream significantly decreased wrinkle length and area and improved skin elasticity [156]. Notably, Rutin upregulates BCL‐2 expression and downregulates BAX levels [155], which may inhibit apoptosis of senescent cells and thus limit its senomorphic efficacy. Therefore, combining rutin with senolytic agents may improve therapeutic outcomes. Additionally, high concentrations of rutin have shown cytotoxicity against melanoma cells in vitro [157], highlighting the need for additional safety evaluation for skin applications.

#### 4.1.3 mTOR‐Related Inhibitors

The mechanistic target of rapamycin (mTOR), a serine/threonine protein kinase, is a crucial regulator of the aging process [158]. mTOR enhances the transcription of IL‐1α, which contributes to the expression of the NF‐κB‐mediated SASP [159]. Moreover, the transcription factor GATA4 activates NF‐κB, whereas mTOR inhibits autophagy during senescence, thereby stabilizing GATA4 and further promoting SASP expression [159]. Declining autophagic activity also elevates intracellular ROS, which can accelerate the aging process [160]. Thus, inhibition of mTOR signalling represents a potential strategy to delay aging (Figure 3).

Rapamycin (RAPA), a natural macrolide isolated from Streptomyces species, is a potent inhibitor of mTOR signalling [161, 162]. RAPA reduces expression of SASP factors, including IL‐6 and IL‐8, in human fibroblasts by inhibiting translation of IL‐1α, thereby limiting the tumorigenic potential of senescent cells in vivo [163]. By inhibiting mTOR and thereby enhancing autophagy, RAPA lowers intracellular ROS in primary human skin fibroblasts and alleviates oxidative senescence [164]. Additionally, RAPA can directly activate TGF‐β receptors independently of TGF‐β, initiating the TGF‐β/Smad pathway and promoting collagen expression [165, 166]. Notably, a randomized, prospective exploratory trial conducted by Chung et al. demonstrated that topical application of RAPA could increase collagen content in the dermis and improve the appearance of fine lines and pigmentation [167]. However, the potential side effects of RAPA, including impaired glucose tolerance, diabetes and immunosuppression, require careful consideration [168].

Metformin (MF), an FDA‐approved antidiabetic agent, also exhibits anti‐aging properties and has been reported to extend lifespan [169, 170, 171, 172]. MF activates AMPK, which inhibits the mTOR signalling pathway via Tuberous Sclerosis Complex 2 (TSC2), a negative regulator of mTOR, thereby attenuating inflammation and delaying the aging process [173]. AMPK activation by MF also promotes macrophage polarization toward the anti‐inflammatory M2 phenotype [174]. In aged skin, MF reduces hyperglycemia‐induced oxidative damage to dermal collagen, ameliorates UVA‐induced thinning of the epidermis and dermis, and accelerates wound healing [175, 176, 177]. However, potential adverse effects of MF include gastrointestinal discomfort and electrolyte imbalances, which warrant consideration in clinical use [178].

### 4.2 Stem Cell Therapy

Stem cells play a crucial role in addressing aging within the field of regenerative medicine. Among them, mesenchymal stem cells (MSCs) are notable for their extensive sources and applications [179]. Research on MSCs for combating skin aging has progressed to the clinical trial stage, yielding significant outcomes (Table 1). These stem cells possess self‐renewal capabilities and multilineage differentiation potential, enabling them to generate diverse cell types [179]. Moreover, the secretome derived from MSCs plays a central role in combating skin aging.

**TABLE 1:** Recent clinical trials investigating stem cell preparations for skin aging.

Preparation	Study type	Delivery method	Treatment effect	Source
Autologous CEFFE	Preliminary clinical study	Intradermal injection of autologous CEFFE	Notable improvement in periorbital skin colour (reduced dark circles) and reduction of periorbital wrinkles	[180]
ADSC secretome	Single‐blind, randomized split‐face clinical trial	Microneedling and fractional CO_2_ laser	Significant improvements in DPAS and wrinkles in both groups; no notable differences between groups at Weeks 4 and 6	[181]
HACS	Prospective, randomized, split‐face study	Microneedling	Significant improvements in wrinkles, elasticity, hydration and pigmentation	[182]
hUMSC‐CM	Randomized controlled split‐face study	Microneedling	Increased skin elasticity, reduced wrinkles and improved pigmentation	[183]
AMSC‐CM	Analytic experimental research	Microneedling	Significant improvement in photoaging	[184]
AF‐MSC‐CM	Split‐face comparative study	Microneedling	Dermal remodelling and increased epidermal thickness	[185]
Protein extracts from the medium of ADSC	Double‐blind, split‐face, randomized, control study	Microneedling	Significant improvement in skin roughness, elasticity and wrinkles	[186]

Abbreviations: AF‐MSC‐CM, amniotic fluid mesenchymal stem cell‐conditioned medium; AMSC‐CM, amniotic membrane stem cell‐conditioned medium; CEFFE, cell‐free fat extract; DPAS, Dermoscopy Photoaging Scale; HACS, adipose tissue stem cell–derived exosome–containing solution; hUMSC‐CM, human umbilical mesenchymal stem cell–conditioned medium.

In vitro studies have shown that MSCs can differentiate into keratinocyte lineages [187, 188, 189, 190]. Thus, MSCs may directly replace senescent cells through proliferation and differentiation, thereby contributing to anti‐aging effects on the skin. One study reported that intradermal transplantation of adipose‐derived MSCs (ADSCs) improved skin wrinkles and enhanced epidermal integrity in rats, effects that were partially attributable to differentiation of ADSCs into epidermal progenitor cells (EPCs) [189]. However, low survival rates of transplanted stem cells may limit therapeutic efficacy in treating skin aging [189, 191]. Innovative biomaterials offer a promising strategy to overcome this limitation: biomaterial scaffolds provide cell adhesion sites, simulate the ECM microenvironment, and establish an optimal niche that enhances transplanted cell survival [192, 193]. Furthermore, allogeneic stem cell transplants may face challenges related to immune rejection.

Cell‐free therapies, which primarily utilize the secretome, demonstrate significant therapeutic potential due to their reduced immunogenicity and enhanced safety profiles [194]. The secretome derived from MSCs is enriched in various growth factors that promote skin rejuvenation [195, 196, 197, 198]. Kim et al. discovered that conditioned media (CM) from human umbilical cord blood‐derived MSCs contains elevated levels of growth differentiation factor 11 (GDF‐11), which promotes the growth, migration, and synthesis of ECM in human dermal fibroblasts [195]. TGF‐β1, another key secretome component, stimulates collagen synthesis while inhibiting its degradation in fibroblasts [196]. Additional growth factors in the secretome—including FGF family members (FGF2, FGF4, FGF6, FGF7), EGF, TGF‐α, PDGF, VEGF and HGF—regulate fibroblast and keratinocyte proliferation, promote angiogenesis and hair growth, and thereby contribute to skin rejuvenation [195, 199, 200, 201, 202]. However, the low stability of growth factors in vivo, along with their rapid diffusion and potential systemic side effects, limit their clinical application [203]. The incorporation of biomaterials, such as glycosaminoglycan (GAG)‐based matrices, can reduce diffusion and degradation of growth factors, thus offering a promising solution [203, 204].

Secretome therapy can downregulate both the expression and activity of MMPs [196, 205]. Human ADSC‐CM reduce MMP‐1 levels through the MAPK/AP‐1 signalling pathway, thereby alleviating UVB‐induced skin aging [205]. Furthermore, Zhang et al. demonstrated that adipose‐derived MSC‐derived EVs (AMSC‐EVs) and human umbilical cord‐derived MSC‐derived EVs (hUMSC‐EVs) inhibit MMP activity by transferring tissue inhibitor of metalloproteinases 1 (TIMP1), thereby reducing the degradation of ECM proteins [206].

The secretome derived from stem cells may exhibit antioxidant activity. EVs derived from induced pluripotent stem cells (iPSC‐EVs) and MSC‐EVs alleviate oxidative stress and associated cellular aging by directly transferring antioxidant proteins, such as peroxiredoxins (PRDX1 and PRDX2) [207]. Moreover, MSC‐derived exosomes protect cells from damage under stress conditions via adaptive regulation of the NRF2 defence system [208]. Indeed, stem cell secretome therapy has been shown to reduce oxidative stress and cellular senescence in the skin, as well as alleviate UVB‐induced photoaging [209, 210, 211].

Furthermore, the secretome may play a pivotal role in regulating inflammation. Two studies have demonstrated that lactate derived from ADSCs can enhance the expression of immunosuppressive gene programmes, including Nr4a1, by inducing histone acetylation (H3K27ac), which subsequently suppresses the pro‐inflammatory response of macrophages [212, 213]. Notably, the anti‐inflammatory effect of lactate may not depend on macrophage polarization toward the M2 phenotype; instead, it may drive macrophages toward a distinct phenotype characterized by a specific gene expression profile that is closely associated with the silencing of pro‐inflammatory genes [212, 213]. Additionally, research has shown that MSC‐EVs can directly induce M2 polarization [214, 215, 216]. For example, miR‐139‐3p derived from MSCs promotes M2 polarization by inhibiting the expression of STAT1, thereby facilitating cardiac repair following acute myocardial infarction [216]. In the skin, exosomes derived from MSCs have been shown to suppress inflammation and significantly promote the healing of diabetic wounds [217].

The secretome has been shown to regulate the levels of cell cycle proteins p21 and p16 [218]. The upregulation of p21 and p16 serves as a significant marker of cellular senescence [218]. Pan et al. reported that conditioned medium from human amniotic mesenchymal stem cells (hAMSCs) reduced p21 and p16 expression in prematurely senescent human dermal fibroblasts [219].

In summary, stem cell therapy promotes cutaneous rejuvenation through multiple mechanisms, primarily via the replacement of senescent cells, the secretion of growth factors, and the enhancement of antioxidant and anti‐inflammatory activities. These promising findings underscore the need to accelerate clinical translation and to develop standardized protocols for therapeutic application. However, prior to clinical implementation, rigorous safety evaluation and control are imperative, particularly with respect to immune rejection, tumorigenic risk and regulatory compliance.

## 5 Delivery Systems for Anti‐Aging Compounds

The skin barrier significantly limits the transdermal permeability of anti‐aging compounds, thereby reducing their therapeutic efficacy. Consequently, effective delivery systems are critical to improving dermal absorption. Nanocarrier systems, particularly lipid‐based nanomaterials, serve as the primary technological platform for this purpose [220].

Liposomes are spherical vesicles composed primarily of phospholipids, distinguished by their excellent biocompatibility, biodegradability and low toxicity [221]. Their optimal particle size facilitates the penetration of drugs into deeper skin layers [222]. Moreover, the molecules encapsulated in liposomes demonstrate enhanced stability and are more efficiently absorbed by the skin [223]. These characteristics render liposomes effective carriers for anti‐aging compounds. Zhang et al. significantly enhanced the uptake of resveratrol in skin cells by encapsulating it in nanoliposomes (NLP), resulting in improved anti‐wrinkle and whitening effects [224].

The instability of liposomes can lead to drug leakage. Modifying the surface of liposomes or encapsulating them within other carriers, such as hydrogels, double emulsions, or microspheres, can substantially improve their stability [225]. Indeed, Wu et al. demonstrated that the liposome–hydrogel system prevents the rapid clearance of liposomes and enhances the protective efficacy of ascorbyl glucoside (AA2G) against skin photoaging [226]. Additionally, other lipid nanoparticles, including solid lipid nanoparticles (SLN) and nanostructured lipid carriers (NLC), exhibit good stability while providing photoprotection, making them superior alternatives as drug carriers [227].

Due to their composition and particle size, liposomes and solid lipid nanoparticles (SLN) may induce toxicity and undesirable side effects, such as inflammation and skin irritation [228]. In contrast, MSC‐EVs can mitigate these adverse effects while offering a novel delivery method for anti‐aging compounds [228]. EVs can simultaneously deliver both endogenous active ingredients from MSCs and exogenous drugs, facilitating dual loading and significantly enhancing therapeutic efficacy. Additionally, this approach reduces the effective therapeutic dose of natural compounds, thereby lowering toxicity [229]. A recent study demonstrated that combining anti‐aging drugs with EVs produced stronger antioxidant effects in human skin compared to single anti‐aging agents (e.g., NAD+, NR and resveratrol) or EVs derived from ADSCs [230].

However, EVs derived from stem cells encounter considerable challenges, such as low retention rates and stability concerns [192]. Encapsulation within a hydrogel matrix offers a promising approach to addressing these issues [192, 231]. Moreover, the high costs involved in the isolation, purification, and large‐scale production of exosomes hinder their broad application [231].

## 6 Machine Learning in the Discovery of Anti‐Aging Drugs

**FIGURE 5:**
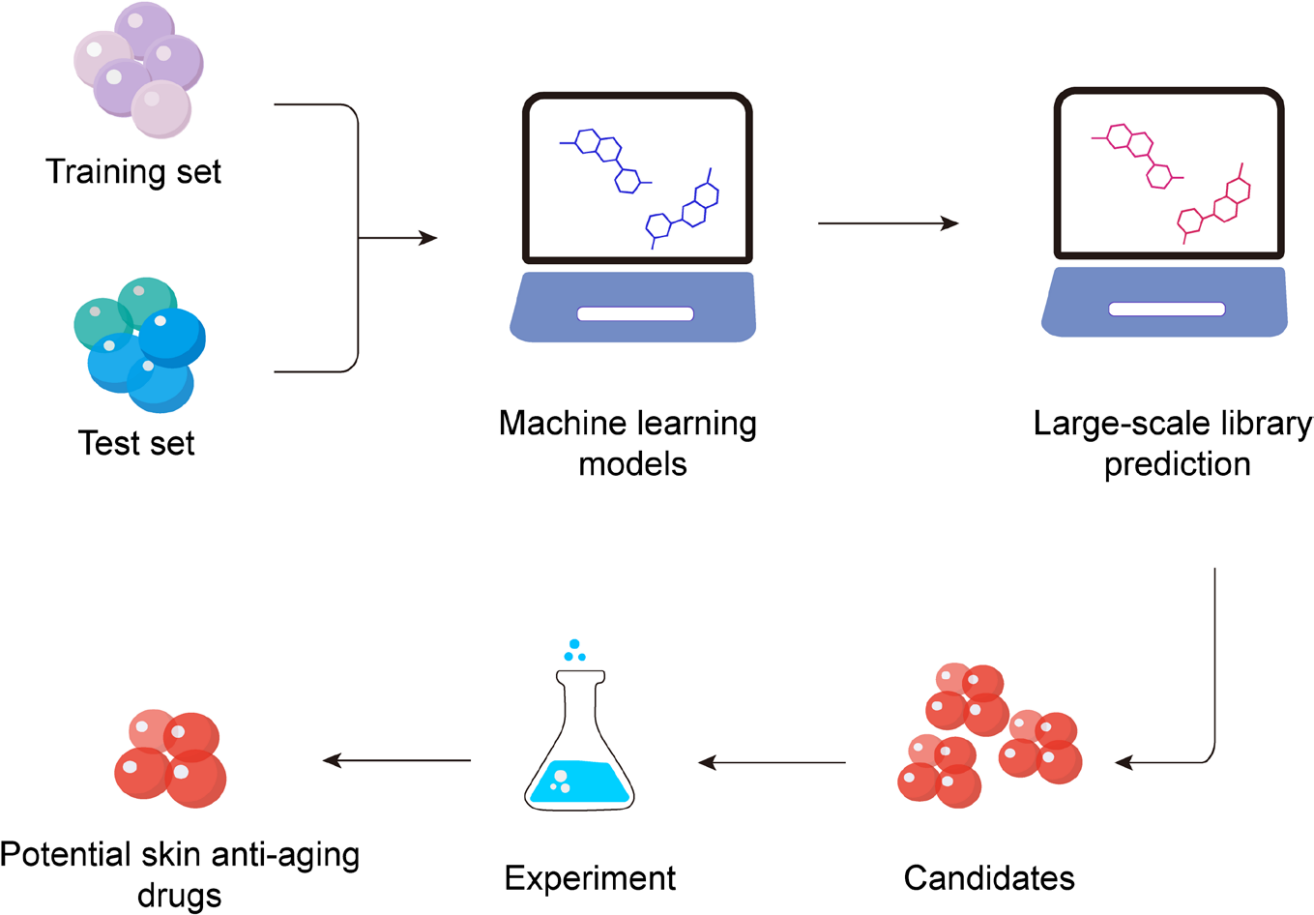
Overview of the machine learning workflow for discovering novel drugs.

The availability of drugs targeting skin aging remains limited, and traditional drug development is time‐consuming, resource‐intensive, and often reliant on iterative trial‐and‐error screening. Consequently, AI‐driven small‐molecule drug discovery has substantially accelerated therapeutic development and holds promise for promoting the rejuvenation of aged skin [232]. Machine learning (ML), a subfield of AI, enables computers to learn from data and to make predictions or decisions without explicit programming [233]. ML models are algorithmic implementations trained on empirical data that can improve their performance as they are exposed to more data [233]. The development of ML models has facilitated the identification of novel anti‐aging drugs (Figure 5).

Smer‐Barreto et al. selected 2523 compounds from public datasets, of which 58 were labelled as senolytic, to train an XGBoost model [234]. Using this model to screen more than 4000 compounds, they identified 21 candidates; subsequent experimental validation confirmed that three compounds—ginkgetin, periplocin and oleandrin—exhibited significant senolytic activity [234]. Similarly, Li et al. developed a Senolytic Predictor that ensembles a support vector machine (SVM) and a multilayer perceptron (MLP) [235]. To increase prediction reliability, only compounds predicted as positive by both models were considered viable candidates. The predictor screened hundreds of compounds from the DrugBank and TCMbank databases, most of which had novel structures, and experimental follow‐up demonstrated that panaxatriol and voclosporin exhibited significant anti‐aging effects [235]. Although these phenotype‐based screening strategies facilitate the identification of candidate anti‐aging agents, they frequently do not elucidate underlying mechanisms of action. Future studies should therefore prioritize characterization of the molecular mechanisms of candidate compounds to uncover novel aging‐related pathways and to inform rational therapeutic design.

## 7 Conclusion and Future Perspectives

Skin aging is a multifactorial biological process characterized by cellular senescence and progressive functional decline across diverse skin cell types. Oxidative stress, DNA damage and epigenetic alterations are major contributors to this process. Senescent cells and the SASP are central targets for anti‐aging interventions and remain the focus of extensive investigation.

Pharmacological approaches are a cornerstone of anti‐aging interventions, yet few candidate agents have progressed to Phase III or later clinical trials [236]. Novel anti‐aging drugs are therefore urgently needed, but traditional drug development is costly and time‐consuming. AI offers promise by mining existing datasets to identify and prioritize candidate bioactive compounds, thereby accelerating early‐stage drug discovery.

The clinical translation of senolytic therapies is limited by cellular heterogeneity, insufficient target specificity, and potential toxicity to non‐senescent cells. Integrating multi‐omics analysis with AI may help resolve aging‐associated cellular heterogeneity and facilitate the design of more precise and effective senolytic strategies, thereby advancing the development of next‐generation precision anti‐aging interventions. The robustness and generalizability of such AI models, however, are dependent on the availability of high‐resolution spatial transcriptomic datasets, which remain limited in the context of human skin aging research. Furthermore, senolytics face pharmacological challenges, including degradation and low bioavailability, which may be mitigated through nanoparticle‐based delivery systems.

Stem cell therapy is a promising strategy for mitigating skin aging. However, it faces several challenges, including immune‐mediated rejection, ectopic differentiation, low post‐transplantation cell survival and engraftment, and the high costs of exosome isolation and production. Innovative biomaterials and emerging technologies—such as 3D bioprinting, gene editing and AI—may help address these limitations.

## Author Contributions

**Sanyin Chen:** methodology, software, data curation, investigation, validation, formal analysis, writing – original draft, writing – review and editing. **Yi Yang:** methodology, software, data curation, investigation. **Xiaohua Pan:** conceptualization, formal analysis, investigation. **Yu Pan:** conceptualization, investigation, supervision, project administration, funding acquisition, writing – review and editing, resources. **Sa Cai:** conceptualization, investigation, supervision, project administration, funding acquisition, writing – review and editing, resources.

## Funding

This work was supported by the National Natural Science Foundation of China (No. 82572914, 82072163), Shenzhen Science and Technology Program (No. JCYJ20240813115701003), Sanming Project of Medicine in Shenzhen Municipality (SZSM202106019) and the 2024 High‐quality Development Research Project of Shenzhen Bao'an Public Hospital (No. BAGZL2024025).

## Conflicts of Interest

The authors declare no conflicts of interest.

## Data Availability

The datasets generated in this article are available from the corresponding authors upon reasonable request.
